# Neuroprotective effects of salvianolic acids combined with Panax notoginseng saponins in cerebral ischemia/reperfusion rats concerning the neurovascular unit and trophic coupling

**DOI:** 10.1002/brb3.70036

**Published:** 2024-09-18

**Authors:** Hongyang Chen, Zhen Liu, Lei Zhao, Zhuangzhuang Jia

**Affiliations:** ^1^ School of Basic Medical Sciences Yunnan University of Chinese Medicine Kunming P. R. China; ^2^ Department of Traditional Chinese Medicine The Baotou Central Hospital Baotou P. R. China; ^3^ State Key Laboratory of Component‐Based Chinese Medicine Tianjin University of Traditional Chinese Medicine Tianjin P. R. China

**Keywords:** ischemic stroke, neurovascular trophic coupling, neurovascular unit, Panax notoginseng saponins, salvianolic acids

## Abstract

**Background:**

The neurovascular unit (NVU) and neurovascular trophic coupling (NVTC) play a key regulatory role in brain injury caused by ischemic stroke. Salvianolic acids (SAL) and Panax notoginseng saponins (PNS) are widely used in China to manage ischemic stroke. Neuroprotective effects of SAL and PNS, either taken alone or in combination, were examined in this research.

**Methods:**

Wistar rats were randomly divided into the following groups: Sham group (Sham), cerebral ischemia/reperfusion group (I/R), I/R with SAL group (SAL), I/R with PNS group (PNS), I/R with SAL combined with PNS (SAL + PNS), and I/R with edaravone group (EDA). Treatment was administered once daily for two days after modeling of middle cerebral artery occlusion/reperfusion (MCAO/R).

**Results:**

Compared with the I/R group, SAL, PNS, or SAL + PNS treatment reduced infarct size, improved neurological deficit score, reduced Evans blue extravasation, increased expression of CD31 and tight junction proteins (TJs), including zonula occludens‐1 (ZO‐1), zonula occludens‐2 (ZO‐2), and junctional adhesion molecule‐1 (JAM‐1). Furthermore, SAL, PNS, or SAL + PNS suppressed the activations of microglia and astrocyte and led to the amelioration of neuron and pericyte injury. Treatment also inhibited NVU dissociation of GFAP/PDGFRβ and Collagen IV/GFAP while upregulated the expression level of BDNF/TrkB and BDNF/NeuN.

**Conclusions:**

SAL and PNS have significantly remedied structural and functional disorders of NVU and NVTC in I/R injury. These effects were more pronounced when SAL and PNS were combined than when used separately.

## Graphical abstract

This study suggests that salvianolic acids and Panax notoginseng saponins can effectively repair cerebral ischemia/reperfusion injury. Specifically, they improve trophic coupling damage and repair the structural and functional damage of neurons, microvascular endothelial cells, microglia, astrocytes, and pericytes in the neurovascular unit, promoting neurovascular unit remodeling. Compared with individual treatment, salvianolic acids combined with Panax notoginseng saponins treatment showed enhanced effects. This treatment method may be a potential strategy for repairing cerebral ischemia/reperfusion injury.

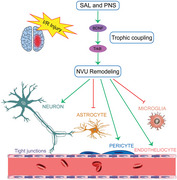

## 1 INTRODUCTION

Globally, ischemic stroke is a leading cause of mortality and morbidity (Walter, 2022). It is accompanied by a series of complex pathophysiological effects, including excitotoxicity, energy metabolism disorders, and apoptosis (Endres et al., 2022; Qin et al., 2022; Zong et al., 2022). Presently, recombinant tissue plasminogen activator (rtPA) represents almost the primary therapeutic choice for ischemic stroke (Chen et al., 2023; Grotta et al., 2021; Mendelson & Prabhakaran, 2021).

Accelerated preservation of the blood–brain barrier (BBB) is a crucial element of every intervention for neuronal damage following a brain injury, as endothelial cells create tight junctions that prevent the dangerous substances and cells to the brain parenchyma (D'Souza et al., 2021). Moreover, current research on cerebrovascular diseases has expanded and highlighted the importance of not only endothelial cells or BBB but also pericytes, astrocytes, neurons, and the extracellular matrix, which together form the NVU (Alarcon‐Martinez et al., 2020; Lyu et al., 2021; Schaeffer & Iadecola, 2021). It is now well understood that the cerebral endothelium is not only an inert network for blood circulation, but it also secretes neuroprotective trophic factors via neurovascular trophic coupling (NVTC) (Guo et al., 2008). In recent years, there has been increased interest in understanding how NVU and NVTC dysfunction affect stroke outcomes.

Extracts mostly from *Salvia miltiorrhiza Bge*. root (Danshen in Chinese) contain salvianolic acids (SAL) (R. N. Wang, Zhao et al., 2021; XD et al., 2019). Ginsenosides Rg1, Rb1, Rd, and Re, as well as notoginsenoside R1, are the primary ingredients of Panax notoginseng saponins (PNS) (Long et al., 2014; Peng et al., 2018; T. Wang et al., 2016). Furthermore, the benefits of SAL and PNS constituents in cerebrovascular disease are widely reported (L. Wang et al., 2023; Xu et al., 2019). Studies have shown that pretreatment with salvianolic acid A has significant improvement effects as compared with autologous thrombus stroke. In vitro endothelial cell experiment, similar intervention methods increase cell viability and TJs degradation after oxygen‐glucose deprivation (OGD) injury (ZO‐1, occludin, claudin‐5) (Liu et al., 2021). In the MCAO/R rat model, intervention with SAL or PNS can significantly reduce the volume of cerebral infarction, inhibit neurological dysfunction, and regulate the polarization level of microglia M1/M2. Similar results have also been validated in ex vivo cell OGD injury experiments (J. Zhang et al., 2021). Nevertheless, the advantages of SAL, as well as PNS for ischemic stroke in regard to NVU as well as NVTC, have still not been thoroughly studied. We used a rodent model of MCAO/R to explore the disease prevention implications of SAL as well as PNS on NVU and NVTC damages.

## 2 MATERIALS AND METHODS

### 2.1 Animals and experimental groups

SPF Wistar rats that weighed between 250 and 280 g were purchased from Vital River company, registration number SCXK (Jing) 2021‐0006. They lived in an environment of 22 ± 2°C with 12/12 h of light and dark. It was easy for the animals to get their chow and water. The animal experiments received permission (TCM‐LAEC2020082) and were conducted experimental research in accordance with the EU Directive 2010/63/EU.

Rats were completely at random assigned: sham group (Sham), cerebral ischemia/reperfusion group (I/R), I/R with SAL group, I/R with PNS group, I/R with salvianolic acids combined with Panax notoginseng saponins group (SAL + PNS), and I/R with edaravone group (EDA). Based on previous research, the optimal dosage and timing of administration have been determined (F. J. Wang et al., 2018). SAL was administered at 21 mg/kg and was provided by Tianjin Tasly company (Cat. No.20180901). PNS was administered at 100 mg/kg and was provided by Wuzhou pharmaceutical company (Cat. No.18080416). EDA was administered at 6 mg/kg as a positive control and was purchased from Nanjing Simcere company (Cat. No.80‐170510). Drugs were administered by intravenous injection after MCAO/R daily for 2 days. Sham and model groups received isodoses of saline solution for their respective treatments.

### 2.2 MCAO/R model

To induce I/R injury in rats, MCAO/R surgical procedure was carried out as mentioned in the previous section (Longa et al., 1989). In brief, anesthetized rats by inhaling 3% isoflurane and the left common carotid artery, left internal carotid artery (ICA), as well as left external carotid artery (ECA), were all completely separated before the experiment began. Therefore, a nylon suture was progressively threaded from the ECA into the ICA at a depth of approximately 2 cm until it was allowed to reach the origin of the middle cerebral artery (MCA). A gentle retraction of the suture was performed just after 1.5 h of occlusion to permit for reperfusion to take place. The blood arteries were separated in the Sham group without occluding the MCA. The success of the rat MCAO/R model is usually evaluated using the Longa scoring system. The Longa scoring system is a commonly used method to investigate the condition of brain ischemia‐reperfusion injury. This scoring system assigns scores based on the severity of neurological deficits in rats, ranging from 0 to 4. Specifically, the Longa scoring system includes the following items: 0 point: no obvious neurological deficits; 1 point: the rat is unable to fully extend its body but can extend the forelimb on the affected side; 2 points: the rat shows circular drifting during walking but no circling behavior; 3 point: the rat exhibits circular drifting and circling behavior; 4 point: the rat is unable to walk and is in a comatose state. The high or low score represents the severity of neurobehavioral damage. A score of 1–3 indicates successful modeling and subsequent experiments can be conducted.

### 2.3 Evaluation of neurological deficits scores

According to our previous method, modified neurological severity score (mNSS) was tested 2 days after MCAO/R (F. J. Wang et al., 2018). The mNSS includes the motor, sensory, reflex, and balance tests. Behavioral abnormalities and defective reflexes were assigned numerical values on a scale from 0 to 18 (0 = normal and 18 = greatest deficit).

### 2.4 Infarct size measurement

Two days after reperfusion, the animals were put under deep anesthesia before decapitation and harvesting of the brains. A total of six coronal slices of brain tissue were indeed obtained from the rat models (2 mm thickness). The slices were immersed in 2% triphenyltetrazolium chloride (TTC) solution (Cat. No. T8877) and incubated in the dark for 15 min at 37°C on a water bath. The infarct area was estimated by a blinded observer using the following formula: The infarct volume ratio (percent) is equal to (the total infracted area/the total sections area) × 100%.

### 2.5 Histological analysis

The animals were put under deep anesthesia by inhaling 3% isoflurane 2 days after MCAO/R. The rats were perfused using 4% (w/v) paraformaldehyde (PFA) solution via hearts before decapitation and harvesting of the brains. Note that 48 h of fixation in 4% PFA solution, dehydration in gradient ethanol, as well as paraffin embedment were used to preserve the rat's brain. They were then sectioned transversely into 5‐µm sections. To investigate the degree of neuronal damage using Nissl staining. After dewaxed and dehydrated, the paraffin sections were soaked in 1% toluidine blue staining for 1 h. The process of HE staining involved staining the tissue with hematoxylin, which bound to acidic components in the cell nucleus and stained them blue. Then, the tissue was counterstained with eosin, which bound to basic components in the cytoplasm and stained them pink. This dual staining allowed for better visualization and differentiation of different structures within the tissue sample. Histological damage in the penumbra was evaluated by HE and Nissl staining, followed by observation by light microscopy at 200× magnification.

### 2.6 Evaluation of BBB function by Evans blue (EB) extravasation

Four mL/kg of 2% EB (Cat. No. E2129) diluted in 1× PBS solution was administered by tail vein injection 4 h before brain harvesting. PBS solution was therefore injected into the rats' hearts by transcardial perfusion. The brain was removed and treated with 50% trichloroacetic acid solution to visualize EB extravasation as described previously.

### 2.7 Immunohistochemical (IHC) staining

IHC staining investigated the expression of CD31 in the penumbra two days after MCAO/R. Rats were anesthetized before perfusion with 4% PFA in 1× PBS solution for fixation. Tissue blocks were treated with post‐fixed in 4% PFA for 48 h before dehydrated in ascending series of sucrose solution. For blocking intrinsic peroxidase, consecutive 20‐m frozen brain slices were produced and treated with % H_2_O_2_ for 8 min. We next used 1× PBS solution to clean the brain sections, followed by a 30‐min blocking step. The slices were incubated at 4°C overnight with mouse anti‐CD31 at 1:200 (ab24590; Abcam). They were set at 37°C for 30 min with the secondary antibody and then processed by diaminobenzidine substrate solution. After brain slices were stained with hematoxylin and permanently fixed, they were observed under an optical microscope (CKX41; Olympus).

### 2.8 Western blotting

Protein of the penumbra tissues was extracted and quantified. Protein was separated and then transferred onto a membrane. Primary antibodies anti‐ZO‐1 at 1:1000 (sc‐33725; Santa Cruz Biotechnology,), anti‐ZO‐2 at 1:1000 (sc‐515115; Santa Cruz Biotechnology), and anti‐JAM‐1 at 1:1000 (sc‐53623; Santa Cruz B) at 4°C overnight after blocking. The next day, washing to remove unbound antibodies and adding secondary antibodies binding to the specific antibodies. Visualization of protein bands and the expression intensity of each target protein/β‐actin were analyzed.

### 2.9 Single fluorescence immunohistochemistry analysis

The sections were managed for 2 h with 10% dyeing blocking solution prior being permeabilized for 2 h with Triton X‐100 solution. The rat's brain slices were therefore treated with the primary antibodies listed below overnight at 4°C, anti‐NeuN at 1:300 (ab177487; Abcam), anti‐ZO‐1 at 1:100 (sc‐33725; Santa Cruz Biotechnology), anti‐ZO‐2 at 1:100 (sc‐515115; Santa Cruz Biotechnology), anti‐JAM‐1 at 1:100 (sc‐53623; Santa Cruz biotechnology), anti‐GFAP at 1:300 (3670; CST), and anti‐Iba1 at 1:300 (17198; CST). Secondary antibodies were subsequently used for 60 min at 37°C to detect the primary antibodies. DAPI (C1006; Beyotime Biotechnology) was added to slices to stain cell nuclei and the brain slices were incubated for 5 min with fluorescent quencher and imaged by fluorescence microscope (IX73; Olympus). The positive cells and fluorescence intensity were evaluated using Image J software.

### 2.10 Double‐fluorescence immunohistochemistry and vascular dissociation index

Double immunofluorescence staining for N‐acetylglucosamine oligomers (NAGO) plus PDGFRβ, glial fibrillary acidic protein (GFAP) plus PDGFRβ, and GFAP plus collagen IV was conducted to detect the pathological damages to NVU in the penumbra of the ischemic zone. Lycopersicon esculentum lectin (LEL) is a glycoprotein released in vascular endothelial cells that has a favorable affinity for NAGO. Double immunofluorescence staining for BDNF plus NeuN and BDNF plus TrkB confirmed NVTC abnormalities. Types of primary antibodies: anti‐PDGFRβ at 1:200 (ab32570; Abcam), biotinylated LEL at 1:200 (B‐1175; Vector), anti‐GFAP at 1:300 (3670; CST), anti‐collagen IV at 1:400 (ab19808; Abcam), anti‐BDNF at 1:400 (ab108319; Abcam), anti‐NeuN at 1:500 (ab104224; Abcam), and anti‐TrkB at 1:100 (sc‐377218; Santa Cruz). The samples were then evaluated and imaged by laser scanning confocal microscopy (LSM800; Zeiss). Stained cells in the penumbra of ischemia were counted on five randomly selected regions.

Detachment of astrocyte end‐feet from the basement membrane in the GFAP/collagen IV double‐labeled sections or from pericytes in the GFAP/PDGFRβ double‐labeled sections, as well as detachment of pericytes from vascular endothelial cells as assessed by PDGFRβ/NAGO. The area between two kinds of cells of each blood vessel, as well as the length of each blood vessel, was measured. The vascular dissociation index was computed using the area‐to‐length ratio as the input. Using the similar method, both the region between endothelium and pericytes or astrocyte end‐feet, as well as the region between astrocyte end‐feet and pericytes were determined.

### 2.11 Statistical analysis

The experimental data are represented by mean and standard deviation (SD). Analyses of variance with least significant difference (LSD) or the Tamhane's T2 post hoc analysis employing SPSS 20.0 were used to examine intergroup variations in data. *p *< .05 is considered statistically significant.

## 3 RESULTS

### 3.1 SAL and PNS reduce the infarct volume and ameliorate neurological deficits

**FIGURE 1:**
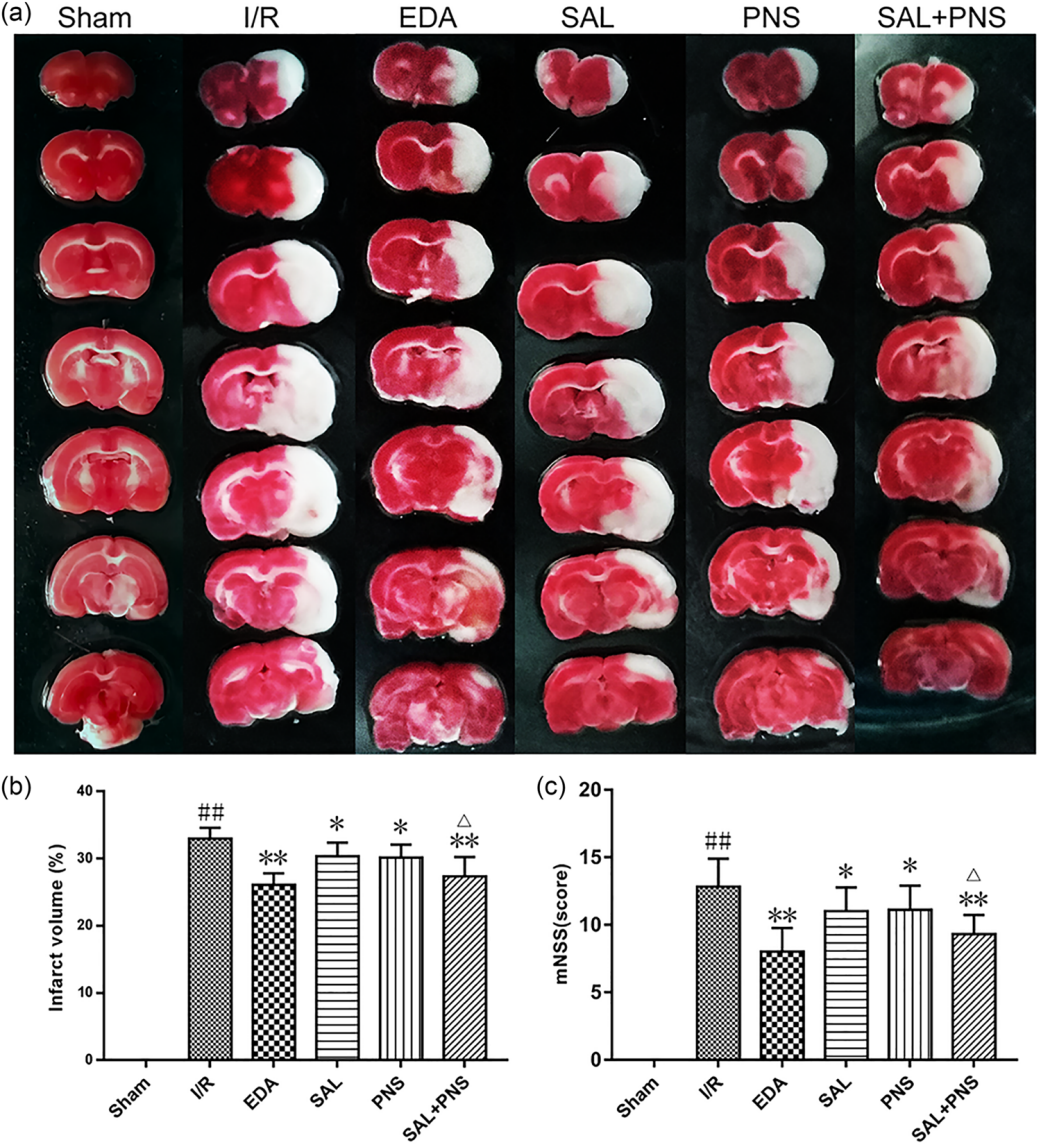
Effect of salvianolic acids (SAL) and Panax notoginseng saponins (PNS) on infarct volume and neurological function in cerebral I/R rats. (A) Representative images of TTC staining. (B) Statistical analysis of infarct volume ratio (*n* = 6 in each group). (C) Statistical analysis of mNSS score at the day 2 (*n* = 10 in each group). Data are presented as mean ± SD. ^##^ *p *< .01 versus Sham; *p < .05, **p < .01 versus I/R; ^△^ *p *< .05 versus SAL or PNS.

As shown in Figure 1A, the ischemic regions of the brains were colored white, while the non‐ischemic region appeared red. Compared with the Sham group, the cerebral infarction size in the I/R group showed a significant upregulation trend. SAL, PNS, SAL+ PNS, and EDA groups were significantly downregulated the cerebral infarction size compared to the model rats (Figure 1B). Neurological deficits analysis revealed that relative to the Sham group, the model rats exhibited clear neurological deficits (Figure 1C). Relative to untreated animals, symptoms were improved upon treatment with SAL, PNS, SAL + PNS, and EDA. Another point to point out was that SAL + PNS treatment had the most marked effect. Combining SAL and PNS therapies significantly reduced the risk of cerebral I/R injuries in the studied group.

### 3.2 Effects of SAL and PNS on morphological injury

**FIGURE 2:**
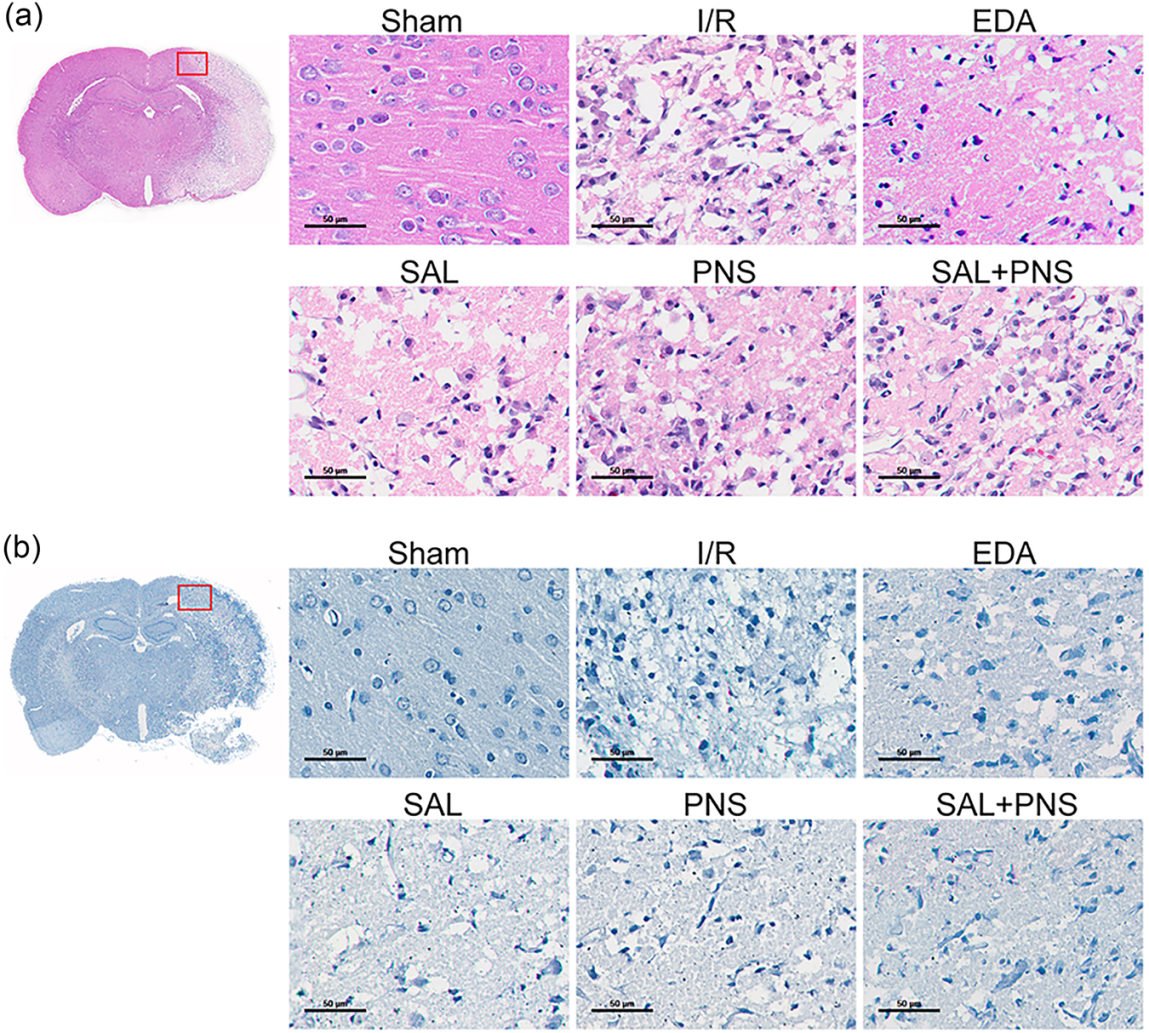
Effect of salvianolic acids (SAL) and Panax notoginseng saponins (PNS) on histological changes of ischemic penumbra in cerebral I/R rats. (A) Histological examination of HE staining (*n* = 5 in each group). (B) Histological examination of Nissl staining (*n* = 5 in each group).

Morphology changes were observed by HE and Nissl staining. These analyses showed that cells of ischemic penumbra in the Sham group had normal morphology. However, neuronal cells of ischemic penumbra in I/R animals displayed altered cell morphology and had pyknotic nuclei (Figure 2A). Nissl staining revealed that relative to neurons of the Sham group animals that had normal morphology and intact structures, those of I/R animals exhibited cytoplasmic disorders, decreased number of Nissl bodies, and nuclear pyknosis (Figure 2B). This damage caused by I/R was ameliorated by treatment with SAL, PNS, SAL + PNS, and EDA. Improvement was most pronounced by combined treatment with SAL + PNS.

### 3.3 Effects of SAL and PNS on BBB function

**FIGURE 3:**
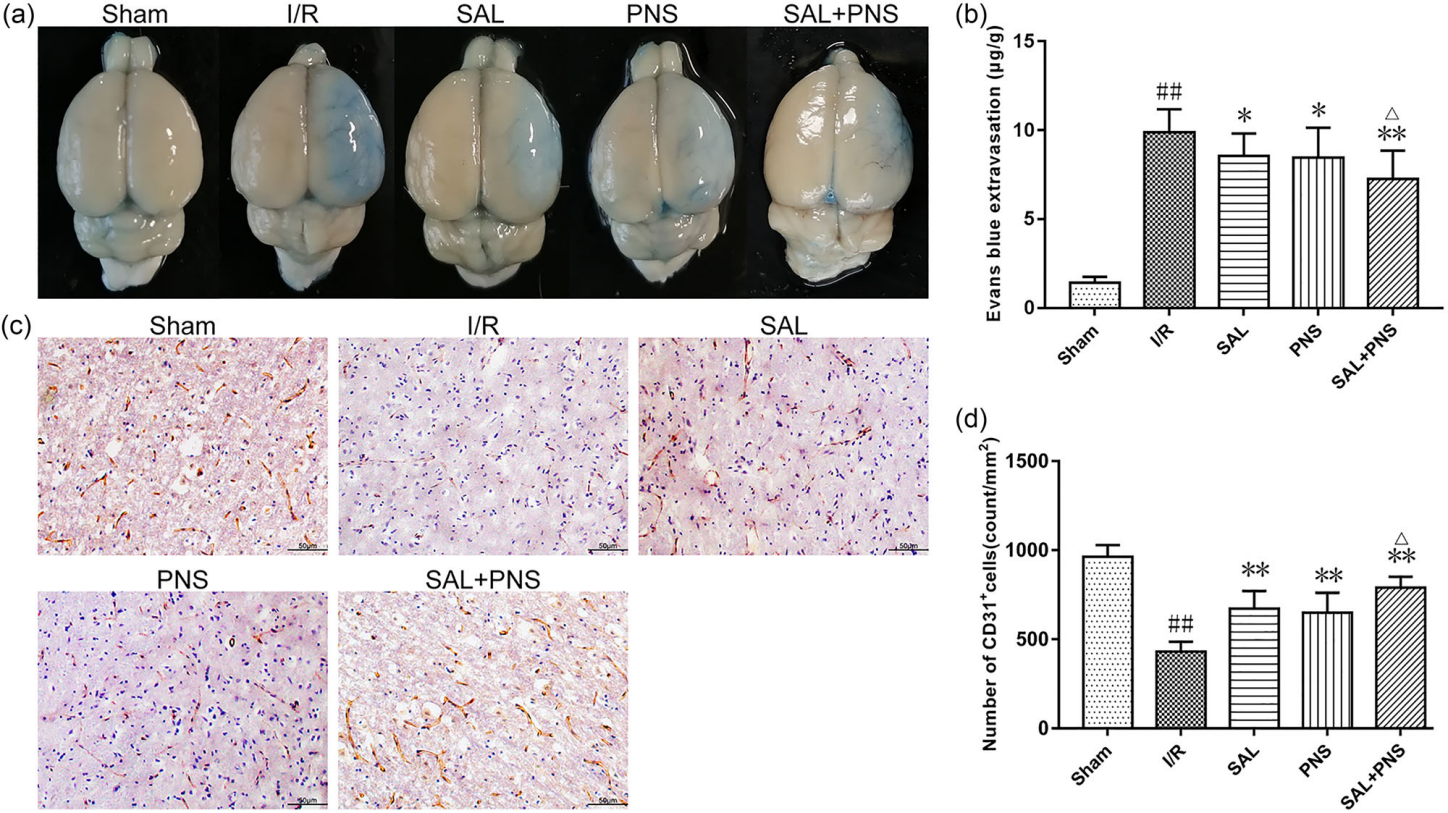
Effects of salvianolic acids (SAL) and Panax notoginseng saponins (PNS) on BBB permeability and CD31 expression in cerebral I/R rats. (A) Representative images of Evans blue staining. (B) Quantitative analysis of Evans blue content in cerebral I/R rats (*n* = 10 in each group). (C) Immunohistochemistry staining showed the positive expression of CD31 in ischemic penumbra. (D) Quantification of the number of CD31 positive cells (*n* = 5 in each group). Data are presented as mean ± SD. ^##^ *p *< .01 versus Sham; *p < .05, **p < .01 versus I/R; ^△^ *p *< .05 versus SAL or PNS.

EB extravasation in I/R injured animals was significantly more severe than in the sham surgery group, as shown in Figure 3A,B. Additionally, treatment with SAL and PNS significantly suppressed EB extravasation. Moreover, the cerebral ischemic penumbra exhibited a marked reduction in CD31 expression following I/R, which was reversed by the administration of SAL and PNS (Figure 3C,D).

**FIGURE 4:**
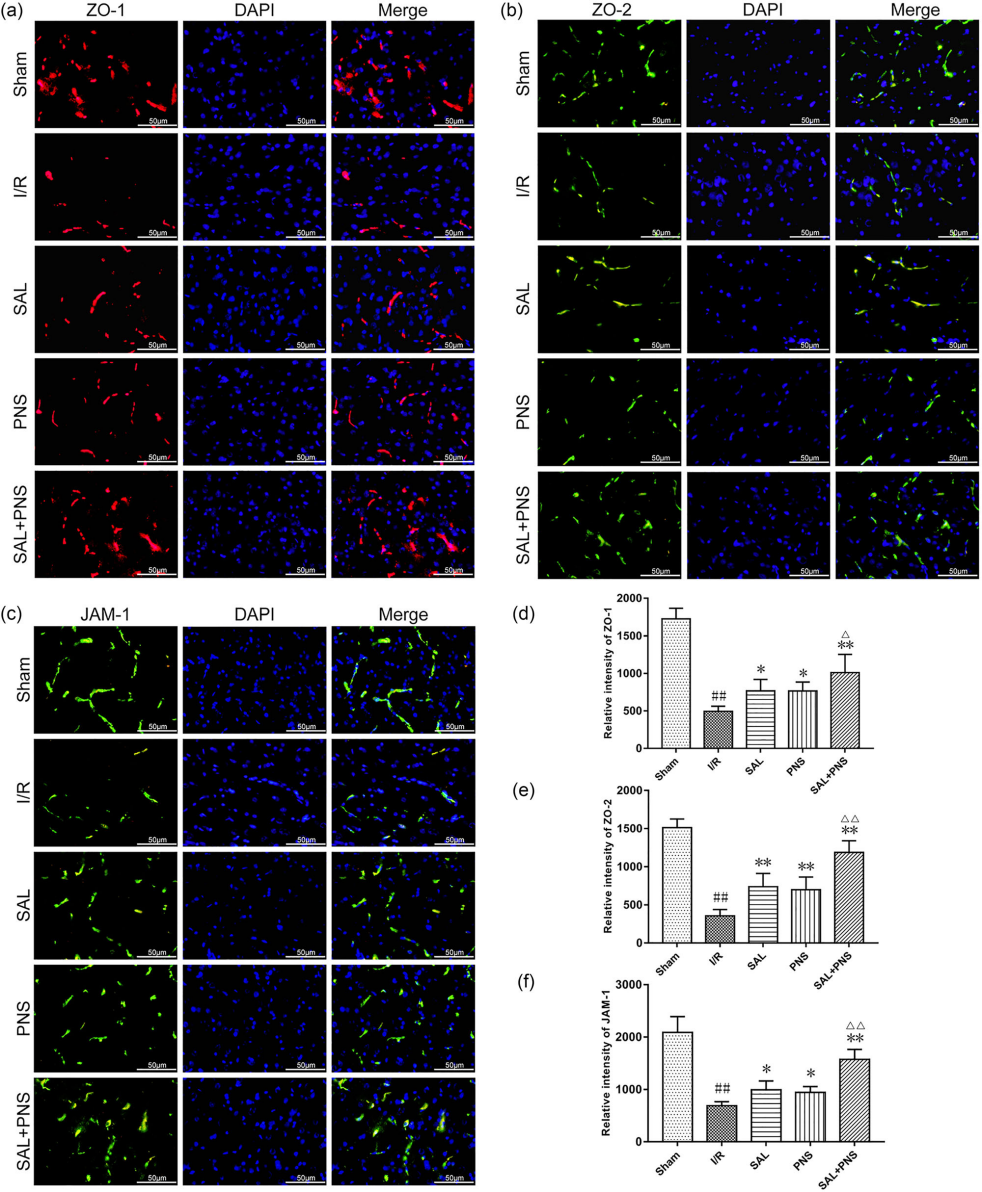
Effect of salvianolic acids (SAL) and Panax notoginseng saponins (PNS) on expression of TJs in cerebral I/R rats. Representative immunofluorescent images of ZO‐1 (A), ZO‐2 (B), and JAM‐1 (C) in ischemic penumbra. Quantification of the relative intensity of ZO‐1 (D), ZO‐2 (E), and JAM‐1 (F) in cerebral I/R rats (*n* = 5 in each group). Data are presented as mean ± SD. ^##^ *p *< .01 versus Sham; *p < .05, **p < .01 versus I/R; ^△^ *p *< .05, ^△△^ *p *< .01 versus SAL or PNS.

**FIGURE 5:**
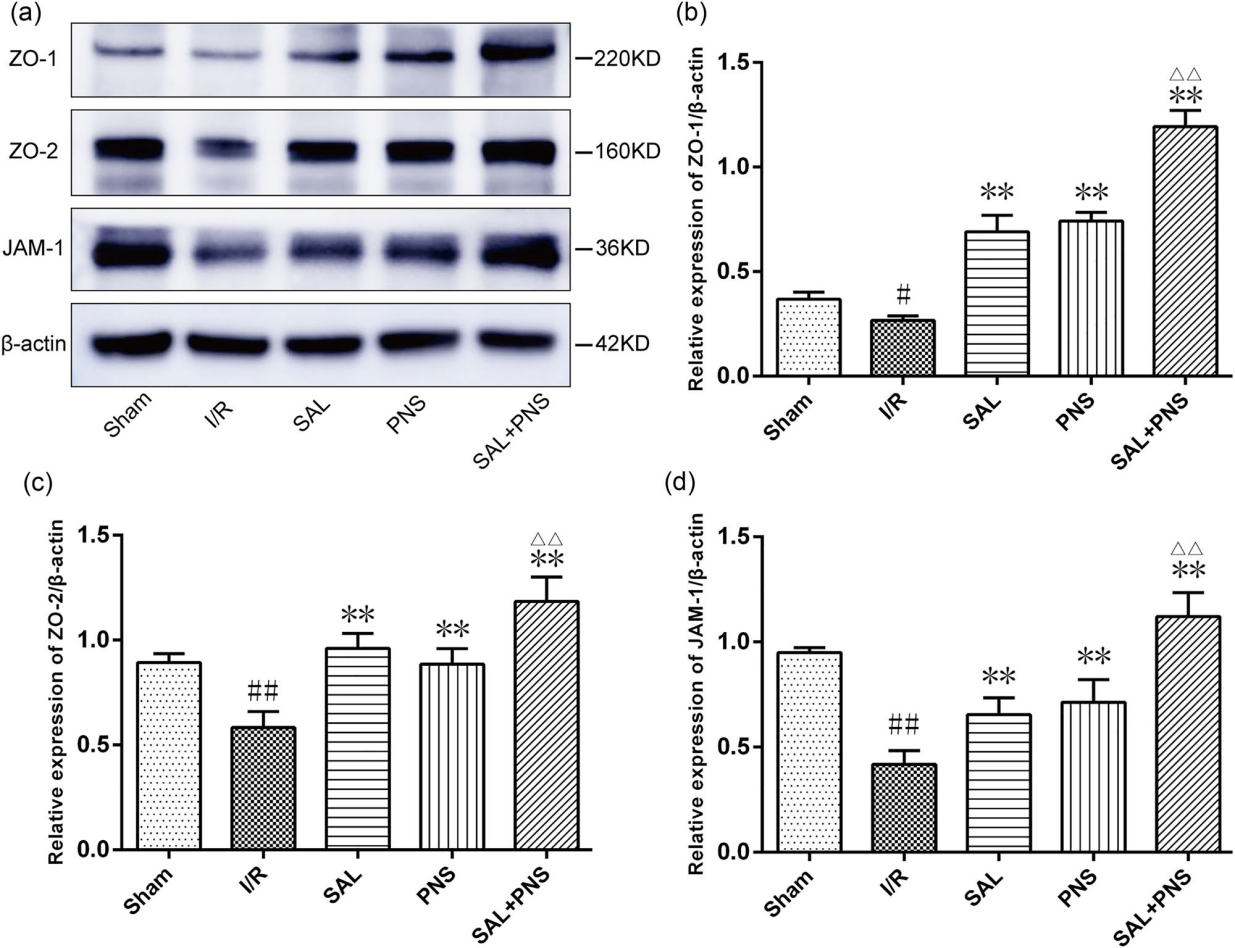
Effect of salvianolic acids (SAL) and Panax notoginseng saponins (PNS) on protein relative expression level of TJs in cerebral I/R rats. Western blot analysis of ZO‐1, ZO‐2, and JAM‐1 in ischemic penumbra, with β‐actin as the loading control (A). Quantification of the relative expression level of ZO‐1 (B), ZO‐2 (C and E), and JAM‐1 (D) in cerebral I/R rats (*n* = 3 in each group). Data are presented as mean ± SD. ^#^ *p *< .05, ^##^ *p *< .01 versus Sham; **p < .01 versus I/R; ^△△^ *p *< .01 versus SAL or PNS.

The results of western blotting and section immunofluorescence staining indicated that the cerebral ischemic penumbra showed a significant decline in ZO‐1, ZO‐2, and JAM‐1 expressions following I/R and administration of SAL and PNS enhanced the expressions of them. These observations were most pronounced upon treatment with SAL + PNS (Figures 4 and 5).

### 3.4 SAL and PNS suppress activations of glial cells in I/R rats

Neuroinflammation damages and an inflammatory response that originates largely from central nervous system (CNS) cell types have been widely established. Chief among these glial cells are microglia and astrocytes. Overactivations of microglia and astrocytes amplify neuroinflammation damage and represent promising targets for stroke management. Staining microglia, as well as astrocytes with immunofluorescence, allowed us to see how SAL and PNS affected their activity. GFAP and microglia‐activated ionized calcium‐binding adaptor molecule 1 (Iba1) are two commonly employed markers for astrocytes and microglia. Glial cells become activated when their number increases and their size and shape change.

**FIGURE 6:**
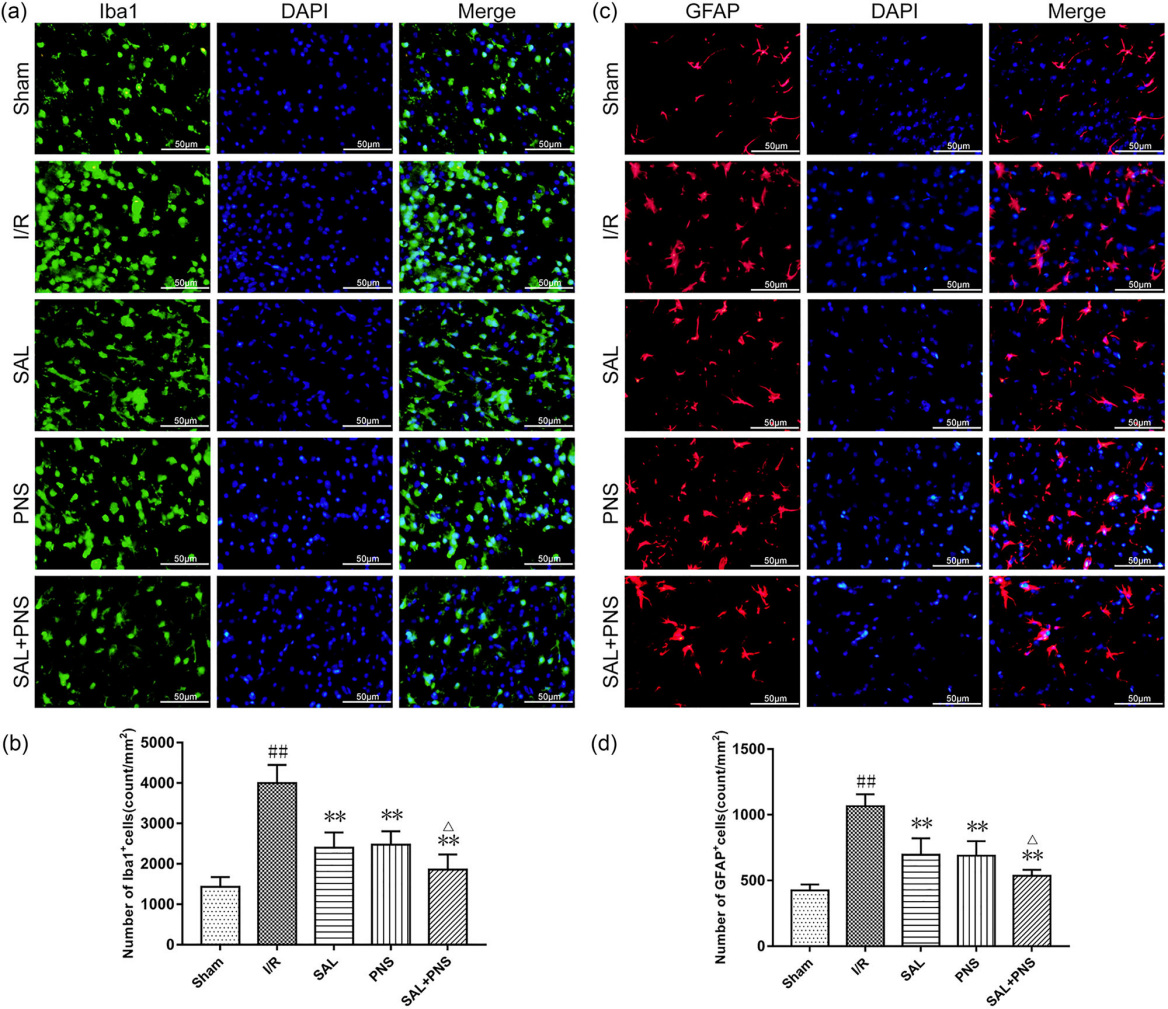
Effect of salvianolic acids (SAL) and Panax notoginseng saponins (PNS) on activations of glial cells in cerebral I/R rats. (A and B) Representative immunofluorescent images and quantification of Iba‐1 in ischemic penumbra. (C and D) Representative immunofluorescent images and quantification of glial fibrillary acidic protein (GFAP) in ischemic penumbra. Data are presented as mean ± SD. ^##^ *p *< .01 versus Sham; **p < .01 versus I/R; ^△^ *p *< .05 versus Salvianolic acids (SAL) or PNS, *n* = 5 in each group.

According to the results prompt in Figure 6, relative to sham surgery animals, the number and size of microglia and astrocytes showed a relatively clear upward trend in the I/R group. Remarkably, treatment with SAL and PNS significantly decreased the number and size of activated microglia and astrocytes compared with the I/R group. This effect was obviously more pronounced in the group treated with SAL + PNS relative to those treated with either SAL or PNS separately, which suggested that SAL and PNS treatments attenuated inflammation of the ischemic brain by inhibiting the activations of microglia and astrocytes.

### 3.5 Effects of SAL and PNS on injuries of neuron and pericyte in I/R rats

**FIGURE 7:**
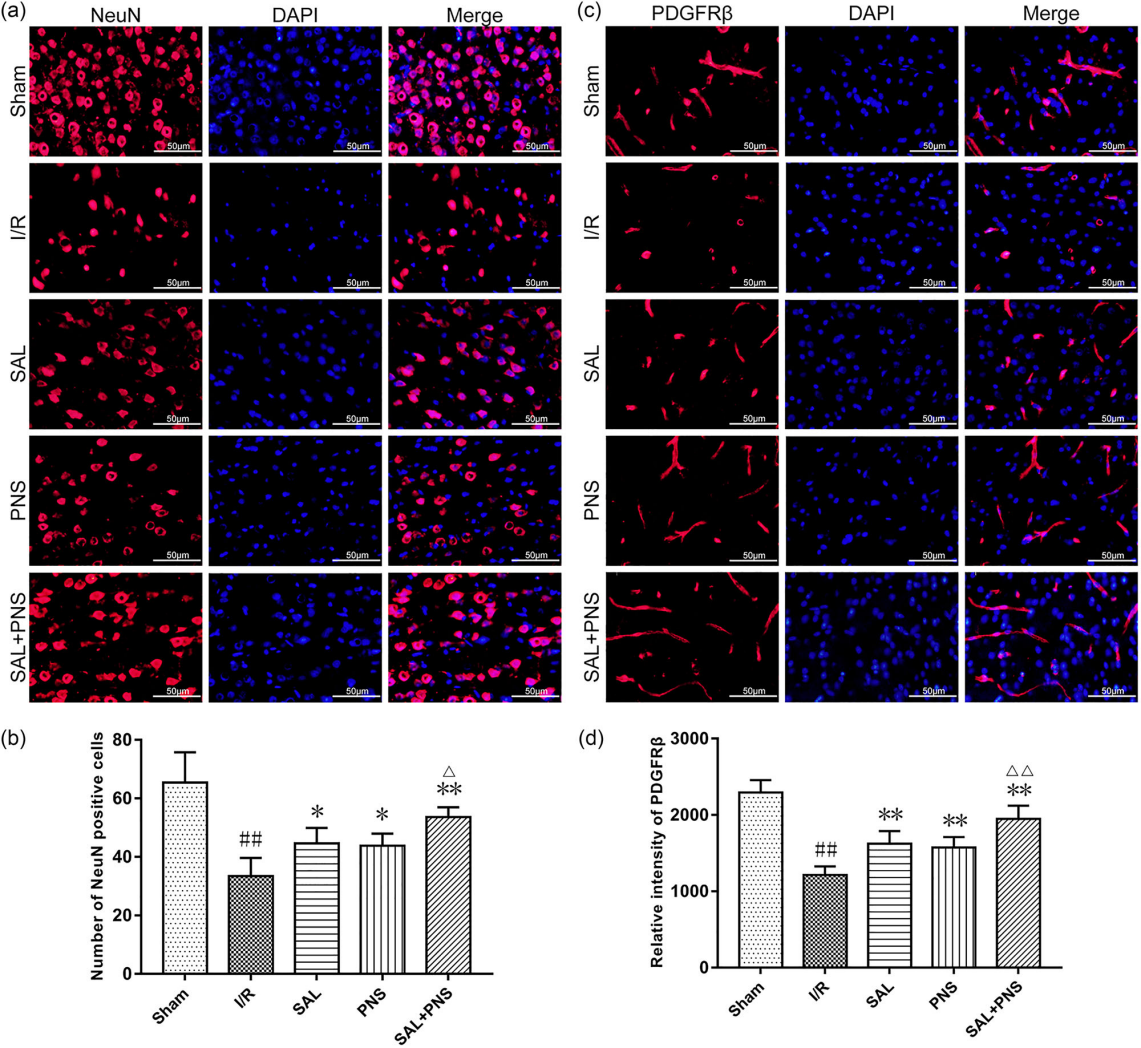
Effect of salvianolic acids (SAL) and Panax notoginseng saponins (PNS) on expression of NeuN and PDGFRβ in cerebral I/R rats. (A and B) Representative immunofluorescent images and quantification of NeuN in ischemic penumbra. (C and D) Representative immunofluorescent images and quantification of PDGFRβ in ischemic penumbra. Data are presented as mean ± SD. ^##^ *p *< .01 versus sham; *p < .05, **p < .01 versus I/R; ^△^ *p *< .05, ^△△^ *p *< .01 versus SAL or PNS, *n* = 5 in each group.

Processes in the brain are already in physical touch with the vasculature, which causes a local elevation in cerebral blood flow (CBF) due to an enhanced neuronal metabolic requirement at that particular region. Also, endothelium and pericyte maintain the function of capillary together. CD31 is an endothelium marker, and platelet‐derived growth factor receptor‐β (PDGFRβ) is used to characterize pericyte. SAL and PNS enhanced the expressions of CD31 (Figure 3). According to the results prompt in Figure 7, relative to the Sham group, I/R injury triggered neuronal reduction and decreased PDGFRβ level. The continuity of pericyte was destroyed in model rats to indicate the integrity of capillary deteriorated. Treatment with SAL, PNS, or both, upregulated the number of neurons along with improved expression and morphological change of the pericyte. These effects were pronounced combined treatment with SAL + PNS than observed with SAL or PNS alone.

### 3.6 SAL and PNS inhibit NVU dissociation

**FIGURE 8:**
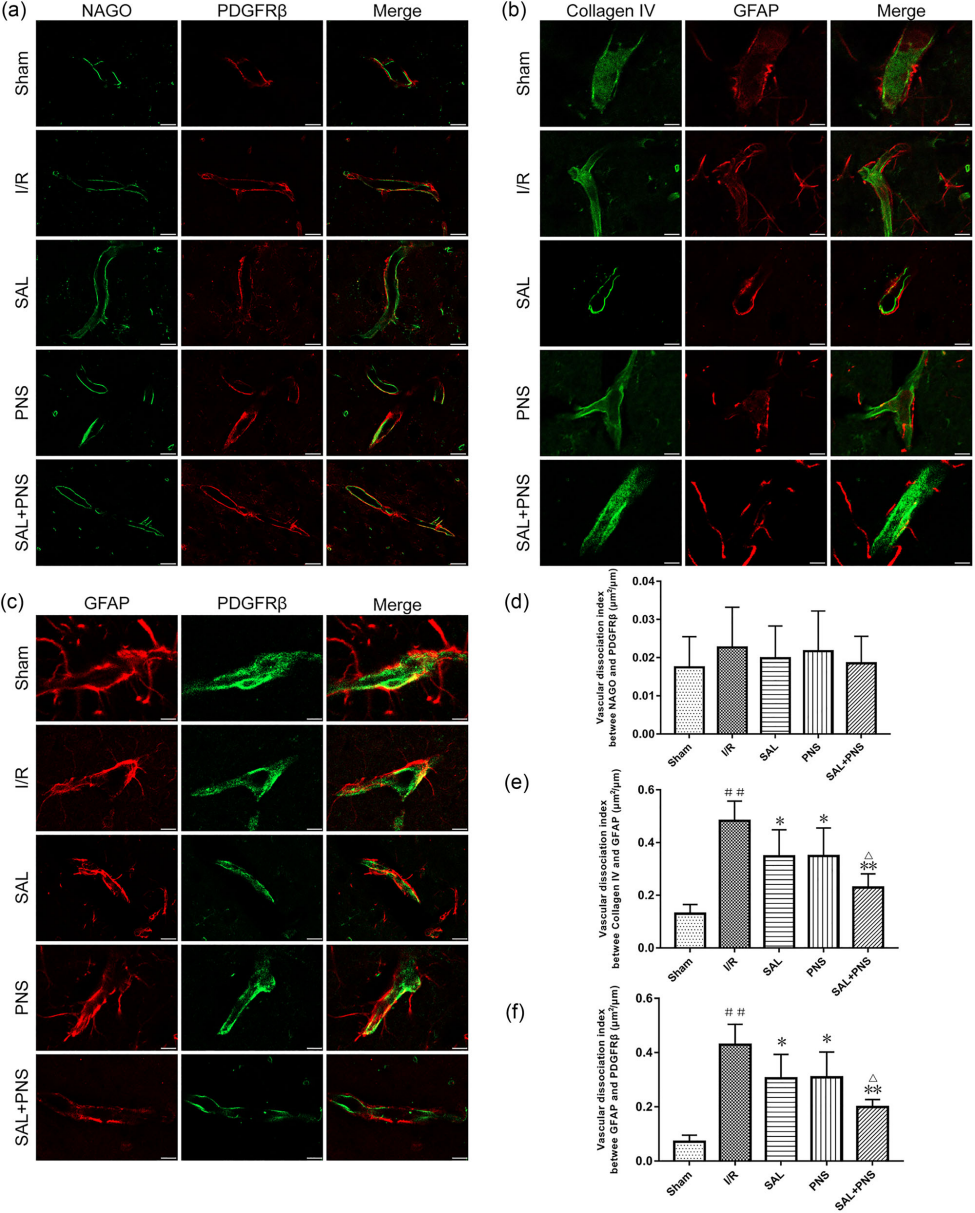
Effect of salvianolic acids (SAL) and Panax notoginseng saponins (PNS) on neurovascular unit (NVU) dissociation in cerebral I/R rats shown by double‐immunofluorescent staining of NAGO/PDGFRβ (A), Collagen IV/GFAP (B) and GFAP/PDGFRβ (C). Quantitative analysis of vascular dissociation index between N‐acetylglucosamine oligomers (NAGO) and PDGFRβ (D), Collagen IV and glial fibrillary acidic protein (GFAP) (E), GFAP and PDGFRβ (F) (scale bar = 20 m). Data are presented as mean ± SD. ^##^ *p *< .01 versus sham; *p < .05, **p < .01 versus I/R; ^△^ *p *< .05 versus SAL or PNS, *n* = 5 in each group.

As shown in Figure 8, vascular dissociation index analysis revealed a marked dissociation of basal lamina (Collagen IV) and astrocyte foot processes (GFAP) in the model rats relative to the sham group. Additionally, dissociation of GFAP and pericytes (PDGFRβ) was considerably higher in the model rats than it was in the sham surgery rats. These effects were dramatically countered by treatment with SAL, PNS, or both, with the combined treatment achieving the most pronounced effect relative to either treatment separately. However, there were no differences in vascular endothelia (NAGO) and PDGFRβ dissociation between the groups.

### 3.7 SAL and PNS ameliorate NVTC dysfunction

**FIGURE 9:**
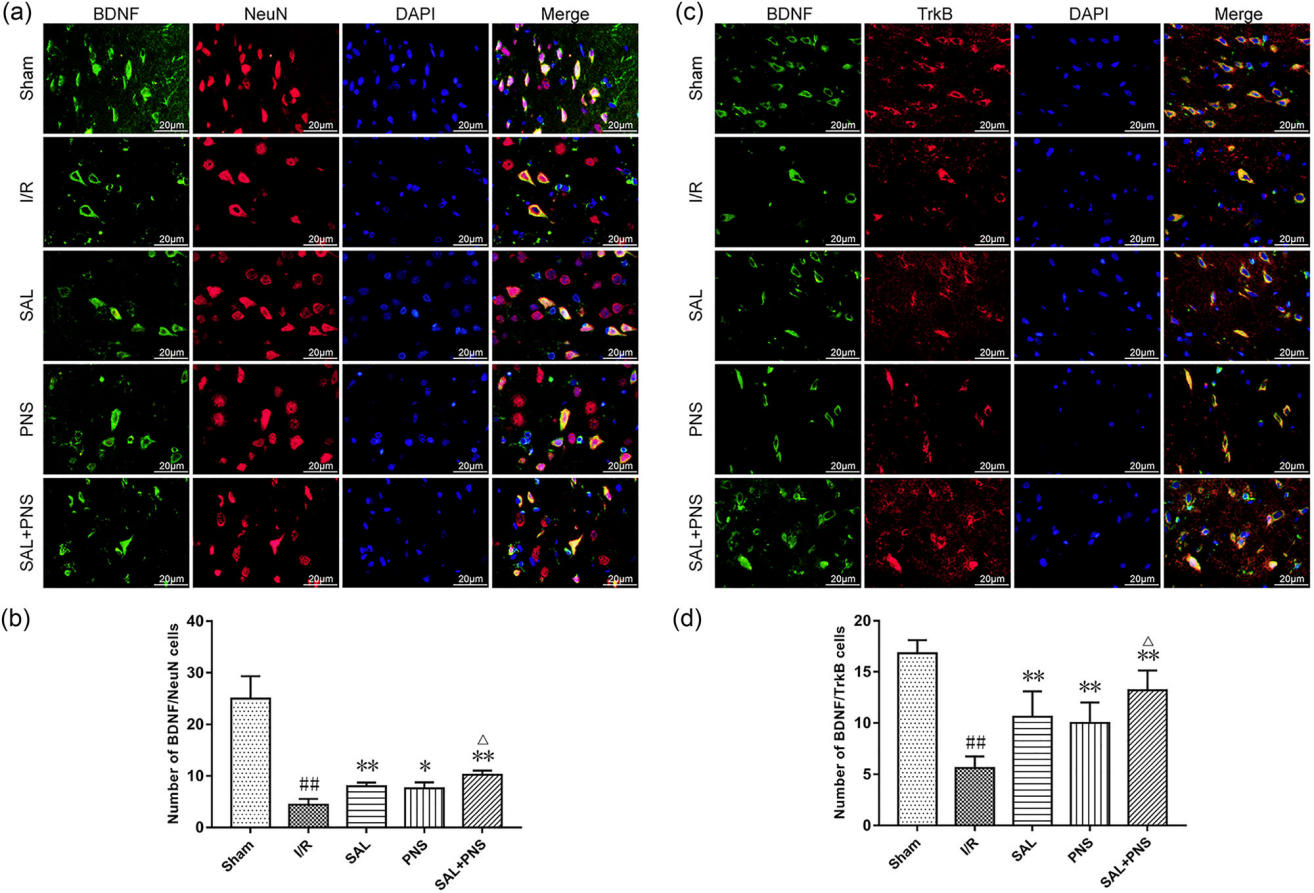
Effect of salvianolic acids (SAL) and Panax notoginseng saponins (PNS) on NVTC dysfunction in cerebral I/R rats shown by double‐immunofluorescent staining. (A and B) Representative immunofluorescent images and quantification of BDNF/NeuN double positive cells in ischemic penumbra. (C and D) Representative immunofluorescent images and quantification of BDNF/TrkB double positive cells in ischemic penumbra. ^##^ *p *< .01 versus sham; *p < .05, **p < .01 versus I/R; ^△^ *p *< .05 versus SAL or PNS, *n* = 5 in each group.

I/R injury decreased double‐fluorescent positive cells of BDNF/NeuN relative to the Sham group. Treatment with SAL, PNS, or both significantly upregulated positive cells of BDNF/NeuN relative to the model rats (Figure 9A,B). A significant reduction in the number of BDNF/TrkB double‐positive cells was observed in the model rats relative to the rats undergoing sham surgery. Treatment with SAL, PNS, and SAL + PNS, an effect was significantly reversed by treatment with SAL, PNS, and SAL + PNS (Figure 9C,D). These observations indicate that the combined therapy with SAL and PNS is ideal for the treatment of NVTC damage.

## 4 DISCUSSION

Ischemic stroke is complicated pathophysiology associated with many pathological changes such as energy failure, oxidative stress, inflammation, and cell apoptosis (Ajoolabady et al., 2021; Tuo et al., 2022). Further study is required in order to develop a better treatment against ischemic stroke (Tao et al., 2020). SAL and PNS comprise multiple bioactive compounds, including lithospermic acid, rosmarinic acid, salvianolic acids (B, E), and ginsenosides (Rg1, Rb1, R1, Re, and Rd). Some of these bioactive constituents exhibit reliable pharmacologic benefits for preventing and treating I/R injury (Feng et al., 2021; L. Wang et al., 2022). Rosmarinic acid has significant neuroprotective, enhancing antioxidant response, anti‐apoptotic, and neuroregenerative effects (Ghasemzadeh Rahbardar & Hosseinzadeh, 2020; J. Wang, Wang, et al., 2021; M. Zhang, Yan, et al., 2017). Ginsenosides have shown significant effects in improving energy metabolism, inhibiting inflammation of microglia, and exhibiting protective effects on primary neurons in vitro (Chen et al., 2015). However, the neuroprotective effects of SAL in combination with PNS against ischemic stroke remain poorly understood. In present study, it was shown that infarct volume, neurobehavioral deficits, and pathological injury were improved by treatment with SAL, PNS, or both.

The BBB is one major component of NVU, comprising an interdependent network of cells (Alahmari, 2021; Kadry et al., 2020). The BBB comprises endotheliocytes, pericytes, astrocytes end‐foot, and basal lamina (Obermeier et al., 2013). Perturbations in BBB integrity are associated with increased vascular permeability and leukocyte infiltration, which may play a pivotal role in ischemic inflammation (Galea, 2021). TJs are located between the endothelial cells and play a pivotal role in controlling brain permeability and homeostasis (Rajagopal et al., 2019). The occludin and claudin families have been shown to be crucial intercellular junction proteins in TJs, which are scaffolded by zonula occludents‐1 (ZO‐1) and zonula occludents‐2 (ZO‐2) (González‐Mariscal et al., 2019; Kuo et al., 2022). Furthermore, JAM‐1 is a member of the immunoglobulin superfamily, which is expressed at TJs in endothelia and displays a crucial role in maintaining the stability of TJs (Zeng et al., 2021). Ischemic stimuli increase BBB permeability and degenerate TJs in the cerebral microvascular endothelial cells (Yang et al., 2022). Salvianolic acid A is the main active component in SAL and has been reported to significantly prevent degradation of Occludin and ZO‐1 to improve BBB in I/R injury (W. Zhang et al., 2018). In this study, administration of SAL and PNS suppressed Evan's blue extravasation. It enhanced increased the expressions of ZO‐1, ZO‐2, and JAM‐1 positive cells, highlighting the potential of SAL and PNS as enhancers of BBB function in the treatment of ischemic cerebral apoplexy.

Glial cells, including astrocyte and microglia, are the most abundant cell types in CNS and participate in NVU formation, which plays a major role in maintaining normal brain function and regulating innate immunity (Ma et al., 2017; Patabendige et al., 2021). Neuroinflammation following ischemic stroke has been documented to disrupt the BBB (Franke et al., 2021). Additionally, they are physically associated with synapses, and their role in glial‐mediated ischemia tolerance and glia‐mediated brain remodeling is mainly unidentified. Astrocytes and microglia are incredibly responsive to alterations in the brain's microenvironment and might get activated quickly and effectively in response (Shinozaki et al., 2017). Reactive gliosis is an essential pathological process following ischemic stroke. The research results indicate that GFAP and Iba1 expression in reactive astrocytes and microglia elevated, an event indicative of reactive gliosis. Results show that treatment with SAL, PNS, or both, suppresses reactive gliosis after I/R injury. Taken together, this suggests that SAL and PNS may exert their protective effect on NVU by regulating reactive gliosis. It has been confirmed that SAL can inhibit programmed cell death of astrocyte after cerebral ischemia/reperfusion injury (Hou et al., 2016). There are also reports that SAL can improve the coverage of pericytes and astrocytes in the peripheral area of cerebral infarction (Li et al., 2017). Moreover, as a pure compound isolated from PNS, gypenosides have a beneficial effect on cognitive impairment by activating astrocytes (X. Wang et al., 2017).

Pericytes locate in around the endothelial cells and embeds in basement membrane outside cerebral microvascular (Sweeney et al., 2016), which have significant roles in cerebral capillary formation and maintenance, BBB formation and stabilization, and regulation of CBF (Hall et al., 2014). Pericyte constricts capillary during cerebral ischemia due to the upstream artery's occlusion. Pericyte‐deficient mice have reduced CBF and oxygen supply for the brain resulting in NVU uncoupling (Kisler et al., 2017). *Salvia miltiorrhiza Bge*. granules increased the expression level of peripheral cell marker PDGFR‐β, CD31, and angiogenesis‐related proteins (M. X. Zhang, Huang, et al., 2022). At the ex vivo cellular level, SAL and PNS promoted the cell viability of pericytes (T. Zhang, Liu, et al., 2022). The data from this study demonstrated that upon I/R injury, treatment with SAL, PNS, or both increased the expression of PDGFRβ, which suggested that treatment with SAL and PNS targeted at preventing pericyte death was beneficial to the maintenance of BBB function after cerebral ischemia.

Anatomically, the NVU emphasizes the unique relationship between brain cells and the cerebral vasculature, drawing attention to their structural and functional interdependence (del Zoppo, 2010). Regulation of NVU integrity plays a critical role in the pathophysiology of ischemic stroke. As the most abundant subtype of glial cells, astrocytes are a key player in the interaction between the cerebral vascular system. Neurons and astrocytes foot processes surround endothelial cells, which interact with neurons through astrocytes (Tiedt et al., 2022). Pericytes are a part of the NVU and function to dilate cerebral vessels, thereby increasing cerebral blood flow after neuronal excitation. Astrocytic foot processes and the basal lamina were shown to be significantly dissociated after I/R injury, which was also the dissociation between pericytes (PDGFR) and these astrocyte foot processes. This dissociation is significantly improved by treatment with SAL and PNS, which was most pronounced by combined treatment with SAL + PNS.

**FIGURE 10:**
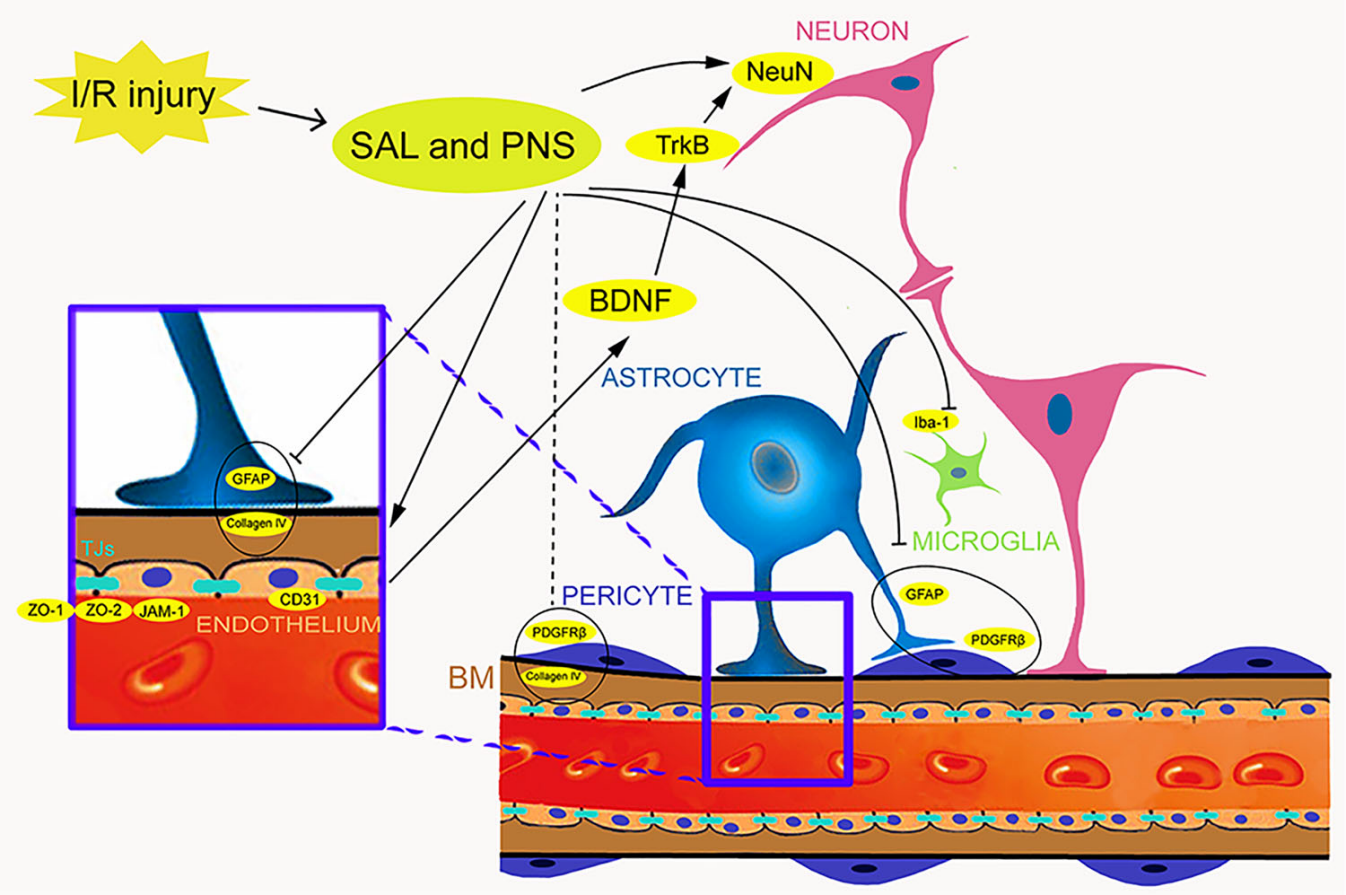
The protective mechanism of salvianolic acids (SAL) and Panax notoginseng saponins (PNS) against cerebral ischemia/reperfusion injury in relation to neurovascular unit and trophic coupling.

Neurotrophic mediators, which help protect neurons from damage and diseases, can be found in the brain's endothelium, which is more than just a supply of inert tubes enabling blood flow to the brain (Di Benedetto et al., 2022). Neuronal impairment might well be mediated by the deterioration of the cerebral endothelium. BDNF is recognized to be derived from brain endothelial cells (Chen et al., 2005). BDNF, as a crucial neurotrophic factor for neuronal structure and function, can have beneficial effects on the development of nerves and synapses by regulating neurotransmitters and endocrine pathways (Luo et al., 2017). Therefore, protecting neurons solely without conserving such crucial endothelium sources of BDNF could be insufficient to achieve good neuroprotective effects. The endothelium no longer produces neuroprotective BDNF, which is bound onto TrkB (Podyma et al., 2021), the primary endogenous receptor of BDNF in neurons, during the process of I/R injury. Previous studies have shown that the BDNF/TrkB signaling pathway and its downstream factors play a positive role in synaptic plasticity (Dietz et al., 2018). After I/R injury, the endothelium appears to lack its trophic supporting capacity for neurons. Interventions with SAL and PNS can inhibit the deficiency of nutritional support of endothelial cells to neurons. SAL can significantly improve brain function after MCAO/R, promotes long‐term survival of newly generated neurons in the subventricular area, accompanied with robust production of BDNF (Y. Zhang, Zhang, et al., 2017). As important components of SAL, cryptotanshinone, and tanshinone IIA can improve brain neural function by regulating BDNF/TrkB signal transduction in the hippocampus (Jiang et al., 2022; K. Wang, Zhai, et al., 2021). PNS can upregulate the generation of BDNF and its downstream signaling molecule TrkB in MCAO rats (Y. Zhang, Zhang, et al., 2022). Importantly, PNS treatment elevated the levels of BDNF and TrkB in the cortex and hippocampus of hypoxic‐ischemic injury rats (Huang et al., 2021). Protective mechanisms of SAL, as well as PNS against cerebral ischemia/ reperfusion injuries in relation to NVU and NVTC, are illustrated in Figure 10.

## 5 CONCLUSION

In summary, study indicates that SAL and PNS are neuroprotective upon ischemic stroke and may significantly ameliorate NVU damage and NVTC function upon I/R injury. Treatment of SAL combined with PNS exhibited an enhanced effect relative to either treatment alone. This treatment approach represents a powerful strategy for protection against focal cerebral ischemia/reperfusion injury.

## AUTHOR CONTRIBUTIONS

**Hongyang Chen**: Methodology; writing—review and editing; writing—original draft; validation; investigation; conceptualization; data curation; visualization. **Zhen Liu**: Methodology; writing—original draft; conceptualization; visualization; data curation. **Lei Zhao**: Conceptualization; methodology; writing—original draft; data curation. **Zhuangzhuang Jia**: Conceptualization; methodology; writing—original draft; writing—review and editing; data curation; investigation; funding acquisition; visualization; project administration; resources.

## CONFLICT OF INTEREST STATEMENT

The authors declare no conflicts of interest.

### PEER REVIEW

The peer review history for this article is available at https://publons.com/publon/10.1002/brb3.70036.

## Data Availability

Please contact the corresponding author to access the data and materials employed or analyzed in this research.

## References

[brb370036-bib-0001] Ajoolabady, A. , Wang, S. , Kroemer, G. , Penninger, J. M. , Uversky, V. N. , Pratico, D. , Henninger, N. , Reiter, R. J. , Bruno, A. , Joshipura, K. , Aslkhodapasandhokmabad, H. , Klionsky, D. J. , & Ren, J. (2021). Targeting autophagy in ischemic stroke: From molecular mechanisms to clinical therapeutics. Pharmacology & Therapeutics, 225, 107848. 33823204 10.1016/j.pharmthera.2021.107848 PMC8263472

[brb370036-bib-0002] Alahmari, A. (2021). Blood‐brain barrier overview: Structural and functional correlation. Neural Plasticity, 2021, 6564585. 10.1155/2021/6564585. 34912450 PMC8668349

[brb370036-bib-0003] Alarcon‐Martinez, L. , Villafranca‐Baughman, D. , Quintero, H. , Kacerovsky, J. B. , Dotigny, F. , Murai, K. K. , & Prat, A. (2020). Interpericyte tunnelling nanotubes regulate neurovascular coupling. Nature, 585, 91–95. 10.1038/s41586-020-2589-x. 32788726

[brb370036-bib-0004] Chen, H. S. , Cui, Y. , Zhou, Z. H. , Dai, Y. J. , Li, G. H. , Peng, Z. L. , Zhang, Y. , Liu, X. D. , Yuan, Z. M. , Jiang, C. H. , Yang, Q. C. , Duan, Y. J. , Ma, G. B. , Zhao, L. W. , Wang, R. X. , Sun, Y. L. , Shen, L. , Wang, E. Q. , Wang, L. H. , … Wang, Y. L. (2023). Effect of argatroban plus intravenous alteplase vs intravenous alteplase alone on neurologic function in patients with acute ischemic stroke: The ARAIS randomized clinical trial. Jama, 329, 640–650. 10.1001/jama.2023.0550. 36757755 PMC9912168

[brb370036-bib-0005] Chen, J. , Zhang, C. , Jiang, H. , Li, Y. , Zhang, L. , Robin, A. , Katakowski, M. , Lu, M. , & Chopp, M. (2005). Atorvastatin induction of VEGF and BDNF promotes brain plasticity after stroke in mice. Journal of Cerebral Blood Flow and Metabolism, 25, 281–290. 10.1038/sj.jcbfm.9600034. 15678129 PMC2804085

[brb370036-bib-0006] Chen, W. , Guo, Y. , Yang, W. , Zheng, P. , Zeng, J. , & Tong, W. (2015). Protective effect of ginsenoside Rb1 on integrity of blood‐brain barrier following cerebral ischemia. Experimental Brain Research, 233, 2823–2831. 10.1007/s00221-015-4352-3. 26070903

[brb370036-bib-0007] Del Zoppo, G. J. (2010). The neurovascular unit in the setting of stroke. Journal of Internal Medicine, 267, 156–171. 10.1111/j.1365-2796.2009.02199.x. 20175864 PMC3001328

[brb370036-bib-0008] Di Benedetto, M. G. , Scassellati, C. , Cattane, N. , Riva, M. A. , & Cattaneo, A. (2022). Neurotrophic factors, childhood trauma and psychiatric disorders: A systematic review of genetic, biochemical, cognitive and imaging studies to identify potential biomarkers. Journal of Affective Disorders, 308, 76–88. 10.1016/j.jad.2022.03.071. 35378148

[brb370036-bib-0009] Dietz, R. M. , Orfila, J. E. , Rodgers, K. M. , Patsos, O. P. , Deng, G. , Chalmers, N. , Quillinan, N. , Traystman, R. J. , & Herson, P. S. (2018). Juvenile cerebral ischemia reveals age‐dependent BDNF‐TrkB signaling changes: Novel mechanism of recovery and therapeutic intervention. Journal of Cerebral Blood Flow and Metabolism, 38, 2223–2235. 10.1177/0271678X18766421. 29611441 PMC6282214

[brb370036-bib-0010] D'souza, A. , Dave, K. M. , Stetler, R. A. , & Manickam, D. S. (2021). Targeting the blood‐brain barrier for the delivery of stroke therapies. Advanced Drug Delivery Reviews, 171, 332–351. 10.1016/j.addr.2021.01.015. 33497734

[brb370036-bib-0011] Endres, M. , Moro, M. A. , Nolte, C. H. , & Dames, C. (2022). Immune pathways in etiology, acute phase, and chronic sequelae of ischemic stroke. Circulation Research, 130, 1167–1186. 10.1161/CIRCRESAHA.121.319994. 35420915

[brb370036-bib-0012] Feng, L. , Han, F. , Zhou, L. , Wu, S. , Du, Y. , Zhang, D. , Zhang, C. , & Gao, Y. (2021). Efficacy and safety of *Panax notoginseng* saponins (Xueshuantong) in patients with acute ischemic stroke (EXPECT) trial: Rationale and design. Frontiers in Pharmacology, 12, 648921. 10.3389/fphar.2021.648921. 33967788 PMC8101545

[brb370036-bib-0013] Franke, M. , Bieber, M. , Kraft, P. , Weber, A. N. R. , Stoll, G. , & Schuhmann, M. K. (2021). The NLRP3 inflammasome drives inflammation in ischemia/reperfusion injury after transient middle cerebral artery occlusion in mice. Brain, Behavior, and Immunity, 92, 223–233. 10.1016/j.bbi.2020.12.009. 33307174

[brb370036-bib-0014] Galea, I. (2021). The blood‐brain barrier in systemic infection and inflammation. Cellular & Molecular Immunology, 18, 2489–2501. 34594000 10.1038/s41423-021-00757-x PMC8481764

[brb370036-bib-0015] Ghasemzadeh Rahbardar, M. , & Hosseinzadeh, H. (2020). Effects of rosmarinic acid on nervous system disorders: An updated review. Naunyn‐Schmiedebergs Archives of Pharmacology, 393, 1779–1795. 10.1007/s00210-020-01935-w. 32725282

[brb370036-bib-0016] González‐Mariscal, L. , Gallego‐Gutiérrez, H. , González‐González, L. , & Hernández‐Guzmán, C. (2019). ZO‐2 is a master regulator of gene expression, cell proliferation, cytoarchitecture, and cell size. International Journal of Molecular Sciences, 20(17), 4128. 10.3390/ijms20174128. 31450555 PMC6747478

[brb370036-bib-0017] Grotta, J. C. , Yamal, J. M. , Parker, S. A. , Rajan, S. S. , Gonzales, N. R. , Jones, W. J. , Alexandrov, A. W. , Navi, B. B. , Nour, M. , Spokoyny, I. , Mackey, J. , Persse, D. , Jacob, A. P. , Wang, M. , Singh, N. , Alexandrov, A. V. , Fink, M. E. , & Saver, J. L. (2021). Prospective, multicenter, controlled trial of mobile stroke units. The New England Journal of Medicine, 385, 971–981. 10.1056/NEJMoa2103879. 34496173

[brb370036-bib-0018] Guo, S. , Kim, W. J. , Lok, J. , Lee, S. R. , Besancon, E. , Luo, B. H. , Stins, M. F. , Wang, X. , Dedhar, S. , & Lo, E. H. (2008). Neuroprotection via matrix‐trophic coupling between cerebral endothelial cells and neurons. PNAS, 105, 7582–7587. 10.1073/pnas.0801105105. 18495934 PMC2396701

[brb370036-bib-0019] Hall, C. N. , Reynell, C. , Gesslein, B. , Hamilton, N. B. , Mishra, A. , Sutherland, B. A. , O'farrell, F. M. , Buchan, A. M. , Lauritzen, M. , & Attwell, D. (2014). Capillary pericytes regulate cerebral blood flow in health and disease. Nature, 508, 55–60. 10.1038/nature13165. 24670647 PMC3976267

[brb370036-bib-0020] Hou, S. , Zhao, M. M. , Shen, P. P. , Liu, X. P. , Sun, Y. , & Feng, J. C. (2016). Neuroprotective effect of salvianolic acids against cerebral ischemia/reperfusion injury. International Journal of Molecular Sciences, 17, 1190. 10.3390/ijms17071190. 27455249 PMC4964559

[brb370036-bib-0021] Huang, J. , Tan, Y. X. , Xue, L. L. , Du, R. L. , Chen, J. J. , Chen, L. , Li, T. T. , Bai, X. , Yang, S. J. , Xiong, L. L. , & Wang, T. H. (2021). Panax notoginseng saponin attenuates the hypoxic‐ischaemic injury in neonatal rats by regulating the expression of neurotrophin factors. European Journal of Neuroscience, 54, 6304–6321. 10.1111/ejn.15428. 34405468

[brb370036-bib-0022] Jiang, Y. L. , Wang, X. S. , Li, X. B. , Liu, A. , Fan, Q. Y. , Yang, L. , Feng, B. , Zhang, K. , Lu, L. , Qi, J. Y. , Yang, F. , Song, D. K. , Wu, Y. M. , & Zhao, M. G. (2022). Tanshinone IIA improves contextual fear‐ and anxiety‐like behaviors in mice via the CREB/BDNF/TrkB signaling pathway. Phytotherapy Research, 36, 3932–3948. 10.1002/ptr.7540. 35801985

[brb370036-bib-0023] Kadry, H. , Noorani, B. , & Cucullo, L. (2020). A blood‐brain barrier overview on structure, function, impairment, and biomarkers of integrity. Fluids and Barriers of the CNS, 17, Article 69. 10.1186/s12987-020-00230-3. 33208141 PMC7672931

[brb370036-bib-0024] Kisler, K. , Nelson, A. R. , Rege, S. V. , Ramanathan, A. , Wang, Y. , Ahuja, A. , Lazic, D. , Tsai, P. S. , Zhao, Z. , & Zhou, Y. (2017). Pericyte degeneration leads to neurovascular uncoupling and limits oxygen supply to brain. Nature Neuroscience, 20, 406–416. 10.1038/nn.4489. 28135240 PMC5323291

[brb370036-bib-0025] Kuo, W. T. , Odenwald, M. A. , Turner, J. R. , & Zuo, L. (2022). Tight junction proteins occludin and ZO‐1 as regulators of epithelial proliferation and survival. Annals of the New York Academy of Sciences, 1514, 21–33. 10.1111/nyas.14798. 35580994 PMC9427709

[brb370036-bib-0026] Li, Y. , Zhang, X. , Cui, L. , Chen, R. , Zhang, Y. , Zhang, C. , & Zhu, X. (2017). Salvianolic acids enhance cerebral angiogenesis and neurological recovery by activating JAK2/STAT3 signaling pathway after ischemic stroke in mice. Journal of Neurochemistry, 143, 87–99. 10.1111/jnc.14140. 28771727

[brb370036-bib-0027] Liu, C. D. , Liu, N. N. , Zhang, S. , Ma, G. D. , Yang, H. G. , Kong, L. L. , & Du, G. H. (2021). Salvianolic acid A prevented cerebrovascular endothelial injury caused by acute ischemic stroke through inhibiting the Src signaling pathway. Acta Pharmacologica Sinica, 42, 370–381. 10.1038/s41401-020-00568-2. 33303991 PMC8027612

[brb370036-bib-0028] Long, W. , Zhang, S. C. , Wen, L. , Mu, L. , Yang, F. , & Chen, G. (2014). *In vivo* distribution and pharmacokinetics of multiple active components from Danshen and Sanqi and their combination via inner ear administration. Journal of Ethnopharmacology, 156, 199–208. 10.1016/j.jep.2014.08.041. 25218322

[brb370036-bib-0029] Longa, E. Z. , Weinstein, P. R. , Carlson, S. , & Cummins, R. (1989). Reversible middle cerebral artery occlusion without craniectomy in rats. Stroke; A Journal of Cerebral Circulation, 20, 84–91. 10.1161/01.STR.20.1.84. 2643202

[brb370036-bib-0030] Luo, J. , Zheng, H. , Zhang, L. , Zhang, Q. , Li, L. , Pei, Z. , & Hu, X. (2017). High‐frequency repetitive transcranial magnetic stimulation (rTMS) improves functional recovery by enhancing neurogenesis and activating BDNF/TrkB signaling in ischemic rats. International Journal of Molecular Sciences, 18(2), 455. 10.3390/ijms18020455. 28230741 PMC5343989

[brb370036-bib-0031] Lyu, Z. , Park, J. , Kim, K. M. , Jin, H. J. , Wu, H. , Rajadas, J. , & Kim, D. H. (2021). A neurovascular‐unit‐on‐a‐chip for the evaluation of the restorative potential of stem cell therapies for ischaemic stroke. Nature Biomedical Engineering, 5, 847–863. 10.1038/s41551-021-00744-7. PMC8524779 34385693

[brb370036-bib-0032] Ma, Y. , Wang, J. , Wang, Y. , & Yang, G. Y. (2017). The biphasic function of microglia in ischemic stroke. Progress in Neurobiology, 157, 247–272. 10.1016/j.pneurobio.2016.01.005. 26851161

[brb370036-bib-0033] Mendelson, S. J. , & Prabhakaran, S. (2021). Diagnosis and management of transient ischemic attack and acute ischemic stroke: A review. Jama, 325, 1088–1098. 10.1001/jama.2020.26867. 33724327

[brb370036-bib-0034] Obermeier, B. , Daneman, R. , & Ransohoff, R. M. (2013). Development, maintenance and disruption of the blood‐brain barrier. Nature Medicine, 19, 1584–1596. 10.1038/nm.3407. PMC4080800 24309662

[brb370036-bib-0035] Patabendige, A. , Singh, A. , Jenkins, S. , Sen, J. , & Chen, R. (2021). Astrocyte activation in neurovascular damage and repair following ischaemic stroke. International Journal of Molecular Sciences, 22(8), 4280. 10.3390/ijms22084280. 33924191 PMC8074612

[brb370036-bib-0036] Peng, M. , Yi, Y. X. , Zhang, T. , Ding, Y. , & Le, J. (2018). Stereoisomers of saponins in *Panax notoginseng* (Sanqi): A review. Frontiers in Pharmacology, 9, 188. 10.3389/fphar.2018.00188. 29593531 PMC5859349

[brb370036-bib-0037] Podyma, B. , Parekh, K. , Güler, A. D. , & Deppmann, C. D. (2021). Metabolic homeostasis via BDNF and its receptors. Trends in Endocrinology and Metabolism, 32, 488–499. 10.1016/j.tem.2021.04.005. 33958275 PMC8192464

[brb370036-bib-0038] Qin, C. , Yang, S. , Chu, Y. H. , Zhang, H. , Pang, X. W. , Chen, L. , Zhou, L. Q. , Chen, M. , Tian, D. S. , & Wang, W. (2022). Signaling pathways involved in ischemic stroke: Molecular mechanisms and therapeutic interventions. Signal Transduction and Targeted Therapy, 7, Article 215. 10.1038/s41392-022-01064-1. 35794095 PMC9259607

[brb370036-bib-0039] Rajagopal, N. , Irudayanathan, F. J. , & Nangia, S. (2019). Computational nanoscopy of tight junctions at the blood‐brain barrier interface. International Journal of Molecular Sciences, 20(22), 5583. 10.3390/ijms20225583. 31717316 PMC6888702

[brb370036-bib-0040] Schaeffer, S. , & Iadecola, C. (2021). Revisiting the neurovascular unit. Nature Neuroscience, 24, 1198–1209. 10.1038/s41593-021-00904-7. 34354283 PMC9462551

[brb370036-bib-0041] Shinozaki, Y. , Shibata, K. , Yoshida, K. , Shigetomi, E. , Gachet, C. , Ikenaka, K. , Tanaka, K. F. , & Koizumi, S. (2017). Transformation of astrocytes to a neuroprotective phenotype by microglia via P2Y(1) receptor downregulation. Cell Reports, 19, 1151–1164. 10.1016/j.celrep.2017.04.047. 28494865

[brb370036-bib-0042] Sweeney, M. D. , Ayyadurai, S. , & Zlokovic, B. V. (2016). Pericytes of the neurovascular unit: Key functions and signaling pathways. Nature Neuroscience, 19, 771–783. 10.1038/nn.4288. 27227366 PMC5745011

[brb370036-bib-0043] Tao, T. , Liu, M. , Chen, M. , Luo, Y. , Wang, C. , Xu, T. , Jiang, Y. , Guo, Y. , & Zhang, J. H. (2020). Natural medicine in neuroprotection for ischemic stroke: Challenges and prospective. Pharmacology & Therapeutics, 216, 107695. 32998014 10.1016/j.pharmthera.2020.107695

[brb370036-bib-0044] Tiedt, S. , Buchan, A. M. , Dichgans, M. , & Lizasoain, I. (2022). The neurovascular unit and systemic biology in stroke—Implications for translation and treatment. Nature Reviews Neurology, 18, 597–612. 10.1038/s41582-022-00703-z. 36085420

[brb370036-bib-0045] Tuo, Q. Z. , Zhang, S. T. , & Lei, P. (2022). Mechanisms of neuronal cell death in ischemic stroke and their therapeutic implications. Medicinal Research Reviews, 42, 259–305. 10.1002/med.21817. 33957000

[brb370036-bib-0046] Walter, K. (2022). What is acute ischemic stroke? Jama, 327, 885. 10.1001/jama.2022.1420. 35230392

[brb370036-bib-0047] Wang, F. J. , Wang, S. X. , Chai, L. J. , Zhang, Y. , Guo, H. , & Hu, L. M. (2018). Xueshuantong injection (lyophilized) combined with salvianolate lyophilized injection protects against focal cerebral ischemia/reperfusion injury in rats through attenuation of oxidative stress. Acta Pharmacologica Sinica, 39, 998–1011. 10.1038/aps.2017.128. 29022576 PMC6256270

[brb370036-bib-0048] Wang, J. , Wang, S. , Guo, H. , Li, Y. , Jiang, Z. , Gu, T. , Su, B. , Hou, W. , Zhong, H. , Cheng, D. , Zhang, X. , & Fang, Z. (2021). Rosmarinic acid protects rats against post‐stroke depression after transient focal cerebral ischemic injury through enhancing antioxidant response. Brain Research, 1757, 147336. 10.1016/j.brainres.2021.147336. 33548269

[brb370036-bib-0049] Wang, K. , Zhai, Q. , Wang, S. , Li, Q. , Liu, J. , Meng, F. , Wang, W. , Zhang, J. , Wang, D. , Zhao, D. , Liu, C. , Dai, J. , Li, C. , Cui, M. , & Chen, J. (2021). Cryptotanshinone ameliorates CUS‐induced depressive‐like behaviors in mice. Translational Neuroscience, 12, 469–481. 10.1515/tnsci-2020-0198. 34900345 PMC8633587

[brb370036-bib-0050] Wang, L. , Liu, Y. , Wei, J. , Liang, X. , & Zhang, Y. (2023). Effects of intravenous thrombolysis with and without salvianolic acids for injection on the functional recovery of patients with acute ischemic stroke: A systematic review, meta‐analysis, and trial sequential analysis. Phytotherapy Research, 37, 2513–2530. 10.1002/ptr.7843. 37092721

[brb370036-bib-0051] Wang, L. , Wang, L. , Wang, H. , & Zhu, T. (2022). Investigation into the potential mechanism and molecular targets of Fufang Xueshuantong capsule for the treatment of ischemic stroke based on network pharmacology and molecular docking. Frontiers in Pharmacology, 13, 949644. 10.3389/fphar.2022.949644. 36188543 PMC9524248

[brb370036-bib-0052] Wang, R. N. , Zhao, H. C. , Huang, J. Y. , Wang, H. L. , Li, J. S. , Lu, Y. , & Di, L. Q. (2021). Challenges and strategies in progress of drug delivery system for traditional Chinese medicine *Salviae Miltiorrhizae Radix et Rhizoma* (Danshen). Chinese Herbal Medicines, 13, 78–89. 10.1016/j.chmed.2020.08.001. 36117766 PMC9476708

[brb370036-bib-0053] Wang, T. , Guo, R. , Zhou, G. , Zhou, X. , Kou, Z. , Sui, F. , Li, C. , Tang, L. , & Wang, Z. (2016). Traditional uses, botany, phytochemistry, pharmacology and toxicology of *Panax notoginseng* (Burk.) F. H. Chen: A review. Journal of Ethnopharmacology, 188, 234–258. 10.1016/j.jep.2016.05.005. 27154405

[brb370036-bib-0054] Wang, X. , Yang, L. , Yang, L. , Xing, F. , Yang, H. , Qin, L. , Lan, Y. , Wu, H. , Zhang, B. , Shi, H. , Lu, C. , Huang, F. , Wu, X. , & Wang, Z. (2017). Gypenoside IX Suppresses p38 MAPK/Akt/NFκB signaling pathway activation and inflammatory responses in astrocytes stimulated by proinflammatory mediators. Inflammation, 40, 2137–2150. 10.1007/s10753-017-0654-x. 28822019

[brb370036-bib-0055] Xd, M. E. , Cao, Y. F. , Che, Y. Y. , Li, J. , Shang, Z. P. , Zhao, W. J. , Qiao, Y. J. , & Zhang, J. Y. (2019). Danshen: A phytochemical and pharmacological overview. Chinese Journal of Natural Medicines, 17, 59–80. 30704625 10.1016/S1875-5364(19)30010-X

[brb370036-bib-0056] Xu, C. , Wang, W. , Wang, B. , Zhang, T. , Cui, X. , Pu, Y. , & Li, N. (2019). Analytical methods and biological activities of *Panax notoginseng* saponins: Recent trends. Journal of Ethnopharmacology, 236, 443–465. 10.1016/j.jep.2019.02.035. 30802611

[brb370036-bib-0057] Yang, Z. , Huang, C. , Wen, X. , Liu, W. , Huang, X. , Li, Y. , Zang, J. , Weng, Z. , Lu, D. , Tsang, C. K. , Li, K. , & Xu, A. (2022). Circular RNA circ‐FoxO3 attenuates blood‐brain barrier damage by inducing autophagy during ischemia/reperfusion. Molecular Therapy, 30, 1275–1287. 10.1016/j.ymthe.2021.11.004. 34763084 PMC8899525

[brb370036-bib-0058] Zeng, M. , Zhou, H. , He, Y. , Du, H. , Yin, J. , Hou, Y. , Zhu, J. , Zhang, Y. , Shao, C. , Yang, J. , & Wan, H. (2021). Danhong injection enhances the therapeutic effect of mannitol on hemispheric ischemic stroke by ameliorating blood‐brain barrier disruption. Biomedicine & Pharmacotherapy, 142, 112048. 34435588 10.1016/j.biopha.2021.112048

[brb370036-bib-0059] Zhang, J. , Guo, F. , Zhou, R. , Xiang, C. , Zhang, Y. , Gao, J. , Cao, G. , & Yang, H. (2021). Proteomics and transcriptome reveal the key transcription factors mediating the protection of Panax notoginseng saponins (PNS) against cerebral ischemia/reperfusion injury. Phytomedicine, 92, 153613. 10.1016/j.phymed.2021.153613. 34500302

[brb370036-bib-0060] Zhang, M. , Yan, H. , Li, S. , & Yang, J. (2017). Rosmarinic acid protects rat hippocampal neurons from cerebral ischemia/reperfusion injury via the Akt/JNK3/caspase‐3 signaling pathway. Brain Research, 1657, 9–15. 10.1016/j.brainres.2016.11.032. 27923634

[brb370036-bib-0061] Zhang, M. X. , Huang, X. Y. , Song, Y. , Xu, W. L. , Li, Y. L. , & Li, C. (2022). *Astragalus propinquus* schischkin and *Salvia miltiorrhiza* bunge promote angiogenesis to treat myocardial ischemia via Ang‐1/Tie‐2/FAK pathway. Frontiers in Pharmacology, 13, 1103557. 10.3389/fphar.2022.1103557. 36699092 PMC9868545

[brb370036-bib-0062] Zhang, T. , Liu, W. , Yang, J. , Xu, H. , Cao, Y. , Guo, L. , Sun, J. , Liang, B. , Du, X. , Chai, L. , Yuan, Q. , & Hu, L. (2022). Components of *Salvia miltiorrhiza* and *Panax notoginseng* protect pericytes against OGD/R‐induced injury via regulating the PI3K/AKT/mTOR and JNK/ERK/P38 signaling pathways. Journal of Molecular Neuroscience, 72, 2377–2388. 10.1007/s12031-022-02082-y. 36394713

[brb370036-bib-0063] Zhang, W. , Song, J. K. , Zhang, X. , Zhou, Q. M. , He, G. R. , Xu, X. N. , Rong, Y. , Zhou, W. X. , & Du, G. H. (2018). Salvianolic acid A attenuates ischemia reperfusion induced rat brain damage by protecting the blood brain barrier through MMP‐9 inhibition and anti‐inflammation. Chinese Journal of Natural Medicines, 16, 184–193. 10.1016/S1875-5364(18)30046-3. 29576054

[brb370036-bib-0064] Zhang, Y. , Zhang, T. , Jia, J. , Jin, C. , & Li, Y. (2022). Analysis of differential gene expression profiles uncovers mechanisms of Xuesaitong injection against cerebral ischemia‐reperfusion injury. Phytomedicine, 103, 154224. 10.1016/j.phymed.2022.154224. 35691081

[brb370036-bib-0065] Zhang, Y. , Zhang, X. , Cui, L. , Chen, R. , Zhang, C. , Li, Y. , He, T. , Zhu, X. , Shen, Z. , Dong, L. , Zhao, J. , Wen, Y. , Zheng, X. , & Li, P. (2017b). Salvianolic Acids for Injection (SAFI) promotes functional recovery and neurogenesis via sonic hedgehog pathway after stroke in mice. Neurochemistry International, 110, 38–48. 10.1016/j.neuint.2017.09.001. 28887094

[brb370036-bib-0066] Zong, P. , Feng, J. , Yue, Z. , Li, Y. , Wu, G. , Sun, B. , He, Y. , Miller, B. , Yu, A. S. , Su, Z. , Xie, J. , Mori, Y. , Hao, B. , & Yue, L. (2022). Functional coupling of TRPM2 and extrasynaptic NMDARs exacerbates excitotoxicity in ischemic brain injury. Neuron, 110, 1944–1958.e8. 10.1016/j.neuron.2022.03.021. 35421327 PMC9233078

